# Supplementary material for: aRgus: Multilevel visualization of non-synonymous single nucleotide variants & advanced pathogenicity score modeling for genetic vulnerability assessment
Source: Comput Struct Biotechnol J. 2023 Jan 25;21:1077–83. doi: 10.1016/j.csbj.2023.01.027 (PMC9900257; doi:10.1016/j.csbj.2023.01.027)
Supplement: Supplementary file 1 — Supplementary material [file mmc1.docx]

1. **Supplementary files**
   1. **Figure S1**


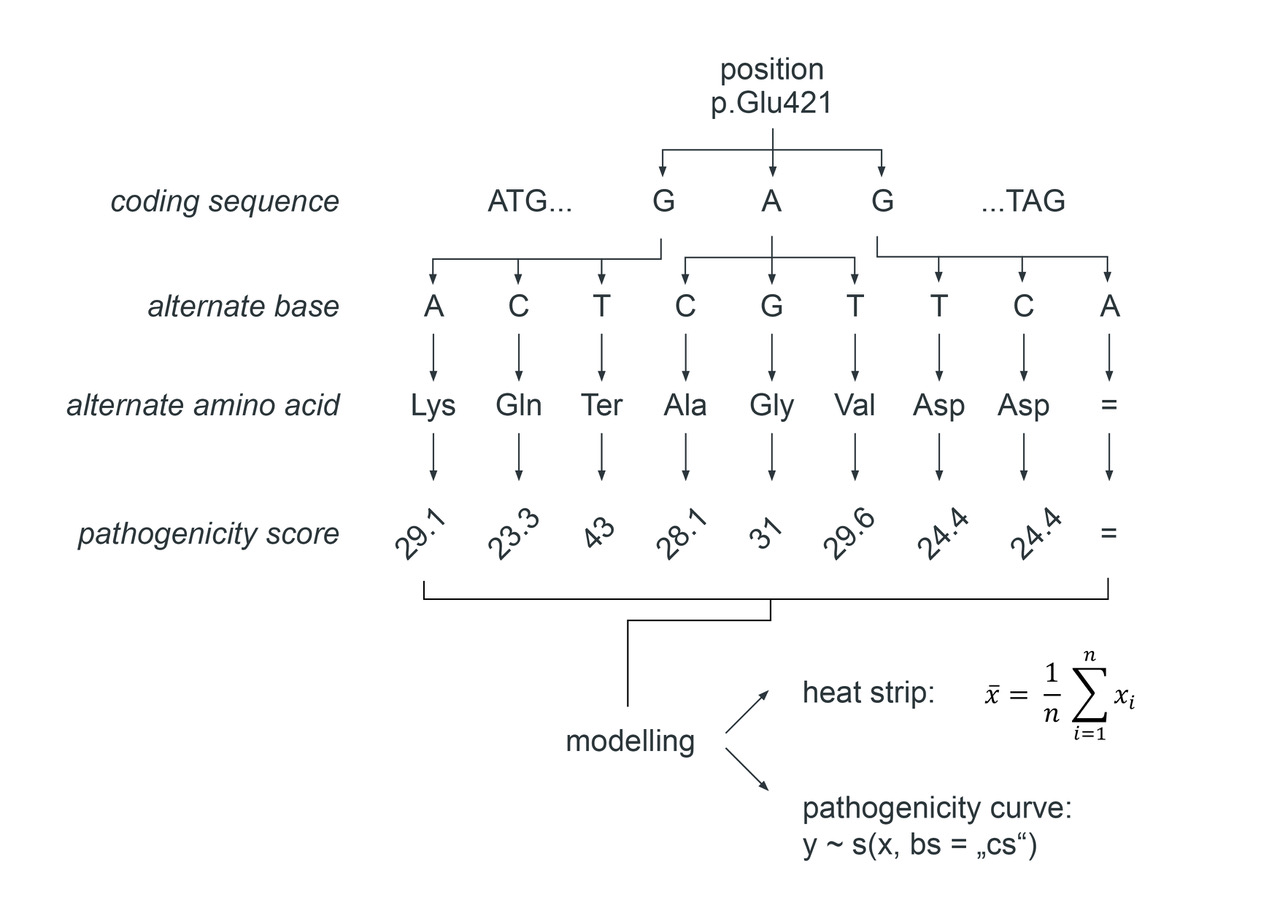


**Fig. S1: Schematic illustration of dbNSFP-derived variant simulation and aRgus-mediated visualization.** Starting from the coding sequence of a gene transcript, any base at any position is exchanged with its three non-synonymous alternate bases (top). Individual pathogenicity score values (bottom) are assigned to the corresponding amino acid substitutions (middle). In aRgus, the resulting tabular data is modeled and visualized using a dual approach with: a) a heat strip color coding position specific arithmetic means using the depicted formula where $\bar{x}$ is the arithmetic mean at a given amino acid position calculated by the sum of pathogenicity score values ($\sum_{i=1}^{n} x_{i})$ divided by $n$ defined as the total number of score values at the given position. And b), a polynomial regression curve using the geom_smooth function of the ggplot2 package. This function is used to add a smooth curve to a plot by fitting a non-parametric regression model to the data, using a specified smoothing method and kernel, and then plotting the resulting curve, where ‘y’ is defined as the response variable of the regression model that is used to fit the smooth curve and ‘x’ is the predictor variable, and ‘s’ defines the type of smoothing with ‘bs’ specifying the bandwidth of the smoothing kernel, which determines the amount of smoothing applied to the curve. Available options for the ‘bs’ parameter depend on the smoothing method being used (in this case: “cs” = cubic splines).

- 1. **Table S1. Pathogenicity scores available on aRgus**

| **Score** | **Version** | **Source** | **DOI** |
| --- | --- | --- | --- |
| REVEL | Release May 3, 2021 | [*https://sites.google.com/site/revelgenomics/*](https://sites.google.com/site/revelgenomics/) | <https://doi.org/10.1016/j.ajhg.2016.08.016> |
| CADD_phred | v1.6 | [*http://cadd.gs.washington.edu/*](http://cadd.gs.washington.edu/) | [https://doi.org/10.1093/nar/gky1016](http://dx.doi.org/10.1093/nar/gky1016) |
| SIFT | ensembl 66 | [*https://sift.bii.a-star.edu.sg/www/history.html*](https://sift.bii.a-star.edu.sg/www/history.html) | <https://doi.org/10.1093/nar/gkg509> |
| SIFT4G | v2.4 | [*http://sift.bii.a-star.edu.sg/sift4g/public//Homo_sapiens/*](http://sift.bii.a-star.edu.sg/sift4g/public/Homo_sapiens/) | <https://doi.org/10.1038/nprot.2009.86> |
| Polyphen HDIV | v2.2.2 | [*http://genetics.bwh.harvard.edu/pph2/*](http://genetics.bwh.harvard.edu/pph2/) | <https://doi.org/10.1038/nmeth0410-248> |
| Polyphen HVAR | v2.2.2 | [*http://genetics.bwh.harvard.edu/pph2/*](http://genetics.bwh.harvard.edu/pph2/) | <https://doi.org/10.1038/nmeth0410-248> |
| PROVEAN | v1.1 ensembl 66 | [*http://provean.jcvi.org/index.php*](http://provean.jcvi.org/index.php) | <https://doi.org/10.1093/bioinformatics/btv195> |
| M-CAP | v1.3 | [*http://bejerano.stanford.edu/MCAP/*](http://bejerano.stanford.edu/MCAP/) | <https://doi.org/10.1038/ng.3703> |
| VEST4 | v4.0 | [*http://karchinlab.org/apps/appVest.html*](http://karchinlab.org/apps/appVest.html) | <https://doi.org/10.1002/humu.22911> |
| FATHMM | v2.3 | [*http://fathmm.biocompute.org.uk*](http://fathmm.biocompute.org.uk) | <https://doi.org/10.1002/humu.22225>  <https://doi.org/10.1093/bioinformatics/btt182>  <https://doi.org/10.1186/1479-7364-8-11> |
| MetaSVM | n/a | [*doi: 10.1093/hmg/ddu733*](about:blank) | <https://doi.org/10.1186/s13040-017-0126-8>  <https://doi.org/10.1093/hmg/ddu733> |
| MetaLR | n/a | [*doi: 10.1093/hmg/ddu733*](about:blank) | <https://doi.org/10.1093/hmg/ddu733> |
| ClinPred | n/a | [*https://sites.google.com/site/clinpred/home*](https://sites.google.com/site/clinpred/home) | <https://doi.org/10.1016/j.ajhg.2018.08.005> |
| MutationTaster | v2 | [*http://www.mutationtaster.org/*](http://www.mutationtaster.org/) | <https://doi.org/10.1038/nmeth.2890> |
| MutationAssessor | Release 3 | [*http://mutationassessor.org/*](http://mutationassessor.org/) | <https://doi.org/10.1093/nar/gkr407> |
| DANN | n/a | [*https://cbcl.ics.uci.edu/public_data/DANN/*](https://cbcl.ics.uci.edu/public_data/DANN/) | <https://doi.org/10.1093/bioinformatics/btu703> |
| MutPred | v1.2 | [*http://mutpred.mutdb.org/*](http://mutpred.mutdb.org/) | <https://doi.org/10.1002/humu.23258>  <https://doi.org/10.1038/s41467-020-19669-x> |
| MVP | v1.0 | [*https://github.com/ShenLab/missense*](https://github.com/ShenLab/missense) | <https://doi.org/10.1038/s41467-020-20847-0> |
| MPC | Release1 | [*ftp://ftp.broadinstitute.org/pub/ExAC_release/release1/regional_missense_constraint/*](ftp://ftp.broadinstitute.org/pub/ExAC_release/release1/regional_missense_constraint/) | <https://doi.org/10.1101/148353> |
| LRT | Release 11/2009 | [*http://www.genetics.wustl.edu/jflab/lrt_query.html*](http://www.genetics.wustl.edu/jflab/lrt_query.html) accessible via [*http://database.liulab.science/dbNSFP*](http://database.liulab.science/dbNSFP) | <https://doi.org/10.1101/gr.092619.109> |
| Primate AI | n/a | [*https://github.com/Illumina/PrimateAI*](https://github.com/Illumina/PrimateAI) | <https://doi.org/10.1038/s41588-018-0167-z> |
| DEOGEN2 | n/a | [*https://deogen2.mutaframe.com/*](https://deogen2.mutaframe.com/) [*http://babylone.3bio.ulb.ac.be/MutaFrame/*](http://babylone.3bio.ulb.ac.be/MutaFrame/) | <https://doi.org/10.1093/nar/gkx390> |
| BayesDel_addAF | v1 | [*http://fengbj-laboratory.org/BayesDel/BayesDel.html*](http://fengbj-laboratory.org/BayesDel/BayesDel.html) | <https://doi.org/10.1002/humu.23158> |
| BayesDel_noAF | v1 | [*http://fengbj-laboratory.org/BayesDel/BayesDel.html*](http://fengbj-laboratory.org/BayesDel/BayesDel.html) | <https://doi.org/10.1002/humu.23158> |
| fathmm.MKL_coding | v2.3 | [*http://fathmm.biocompute.org.uk/fathmmMKL.htm*](http://fathmm.biocompute.org.uk/fathmmMKL.htm) | <https://doi.org/10.1093/bioinformatics/btv009> |
| fathmm.XF_coding | v2.3 | [*http://fathmm.biocompute.org.uk/fathmm-xf/*](http://fathmm.biocompute.org.uk/fathmm-xf/) | <https://doi.org/10.1093/bioinformatics/btv009> |
| Eigen.raw | v1.1 | [*http://www.columbia.edu/~ii2135/eigen.html*](http://www.columbia.edu/~ii2135/eigen.html) | <https://doi.org/10.1038/ng.3477> |
| Eigen.PC.raw | v1.1 | [*http://www.columbia.edu/~ii2135/eigen.html*](http://www.columbia.edu/~ii2135/eigen.html) | <https://doi.org/10.1038/ng.3477> |
| GenoCanyon | v1.0.3 | [*http://genocanyon.med.yale.edu/index.html*](http://genocanyon.med.yale.edu/index.html) [*https://zhaocenter.org/GenoCanyon_Index.html*](https://zhaocenter.org/GenoCanyon_Index.html) | <https://doi.org/10.1038/srep10576> |
| integrated_fitCons | v1.01 | [*http://compgen.bscb.cornell.edu/fitCons/*](http://compgen.bscb.cornell.edu/fitCons/) | <https://doi.org/10.1038/ng.3196> |
| GM12878_fitCons | v1.01 | [*http://compgen.bscb.cornell.edu/fitCons/*](http://compgen.bscb.cornell.edu/fitCons/) | <https://doi.org/10.1038/ng.3196> |
| H1.hESC_fitCons | v1.01 | [*http://compgen.bscb.cornell.edu/fitCons/*](http://compgen.bscb.cornell.edu/fitCons/) | <https://doi.org/10.1038/ng.3196> |
| HUVEC_fitCons | v1.01 | [*http://compgen.bscb.cornell.edu/fitCons/*](http://compgen.bscb.cornell.edu/fitCons/) | <https://doi.org/10.1038/ng.3196> |
| LINSIGHT | n/a | [*http://compgen.cshl.edu/~yihuang/LINSIGHT/*](http://compgen.cshl.edu/~yihuang/LINSIGHT/) [*https://github.com/CshlSiepelLab/LINSIGHT*](https://github.com/CshlSiepelLab/LINSIGHT) | <https://doi.org/10.1038/ng.3810> |
| GERP++_RS | n/a | [*http://mendel.stanford.edu/SidowLab/downloads/gerp/*](http://mendel.stanford.edu/SidowLab/downloads/gerp/) | <https://doi.org/10.1371/journal.pgen.1008827> |
| phyloP100way_vertebrate | n/a | [*http://hgdownload.soe.ucsc.edu/goldenPath/hg38/phyloP100way/*](http://hgdownload.soe.ucsc.edu/goldenPath/hg38/phyloP100way/) | <https://doi.org/10.1101/gr.3715005>  <https://doi.org/10.1101/gr.097857.109> |
| phyloP30way_mammalian | n/a | [*http://hgdownload.soe.ucsc.edu/goldenPath/hg38/phyloP30way/*](http://hgdownload.soe.ucsc.edu/goldenPath/hg38/phyloP30way/) | <https://doi.org/10.1101/gr.3715005>  <https://doi.org/10.1101/gr.097857.109> |
| phyloP17way_primate | n/a | [*http://hgdownload.soe.ucsc.edu/goldenPath/hg38/phyloP17way/*](http://hgdownload.soe.ucsc.edu/goldenPath/hg38/phyloP17way/) | <https://doi.org/10.1101/gr.3715005>  <https://doi.org/10.1101/gr.097857.109> |
| phastCons100way_vertebrate | n/a | [*http://hgdownload.soe.ucsc.edu/goldenPath/hg38/phastCons100way/*](http://hgdownload.soe.ucsc.edu/goldenPath/hg38/phastCons100way/) | <https://doi.org/10.1101/gr.3715005>  <https://doi.org/10.1101/gr.097857.109> |
| phastCons30way_mammalian | n/a | [*http://hgdownload.soe.ucsc.edu/goldenPath/hg38/phastCons30way/*](http://hgdownload.soe.ucsc.edu/goldenPath/hg38/phastCons30way/) | <https://doi.org/10.1101/gr.3715005>  <https://doi.org/10.1101/gr.097857.109> |
| phastCons17way_primate | n/a | [*http://hgdownload.soe.ucsc.edu/goldenPath/hg38/phastCons17way/*](http://hgdownload.soe.ucsc.edu/goldenPath/hg38/phastCons17way/) | <https://doi.org/10.1101/gr.3715005>  <https://doi.org/10.1101/gr.097857.109> |
| SiPhy_29way_logOdds | n/a | [*https://www.broadinstitute.org/mammals-models/29-mammals-project-supplementary-info*](https://www.broadinstitute.org/mammals-models/29-mammals-project-supplementary-info) | <https://doi.org/10.1093/bioinformatics/btp190> |
| LIST.S2_score | Release: 2019_10 | [*https://precomputed.list-s2.msl.ubc.ca/*](https://precomputed.list-s2.msl.ubc.ca/) | <https://doi.org/10.1093/nar/gkaa288> |

*n/a = not applicable*
